# Are Brain and Cognitive Reserve Shaped by Early Life Circumstances?

**DOI:** 10.3389/fnins.2022.825811

**Published:** 2022-06-16

**Authors:** Susanne R. de Rooij

**Affiliations:** ^1^Epidemiology and Data Science, University of Amsterdam, Amsterdam, Netherlands; ^2^Aging and Later Life, Health Behaviors and Chronic Diseases, Amsterdam Public Health Research Institute, Amsterdam, Netherlands; ^3^Amsterdam Reproduction and Development, Amsterdam, Netherlands

**Keywords:** developmental origins, brain reserve, cognitive reserve, prenatal, postnatal, neurodegenerative disorders, dementia

## Abstract

When growing older, many people are faced with cognitive deterioration, which may even amount to a form of dementia at some point in time. Although neuropathological signs of dementia disorders can often be demonstrated in brains of patients, the degree to which clinical symptoms are present does mostly not accurately reflect the amount of neuropathology that is present. Sometimes existent pathology even goes without any obvious clinical presentation. An explanation for this phenomenon may be found in the concept of reserve capacity. Reserve capacity refers to the ability of the brain to effectively buffer changes that are associated with normal aging processes and to cope with pathological damage. A larger reserve capacity has been suggested to increase resilience against age-associated cognitive deterioration and dementia disorders. Traditionally, a division has been made between brain reserve, which is based on morphological characteristics of the brain, and cognitive reserve, which is based on functional characteristics of the brain. The present review discusses the premises that brain and cognitive reserve capacity are shaped by prenatal and early postnatal factors. Evidence is accumulating that circumstances during the first 1,000 days of life are of the utmost importance for the lifelong health of an individual. Cognitive deterioration and dementia disorders may also have their origin in early life and a potentially important pathway by which the early environment affects the risk for neurodegenerative diseases is by developmental programming of the reserve capacity of the brain. The basic idea behind developmental programming of brain and cognitive reserve is explained and an overview of studies that support this idea is presented. The review is concluded by a discussion of potential mechanisms, synthesis of the evidence and relevance and future directions in the field of developmental origins of reserve capacity.

## Introduction

It is increasingly being recognized that neurodegenerative diseases start many years before clinical symptoms become apparent. That the basis for risk of developing neurodegenerative diseases may be laid in the very earliest stages of life is less well-known. It may also seem like a long shot, literally, as the diagnosis of diseases like Alzheimer’s dementia usually takes place toward the end of life and factors playing 70–80 years before that may not seem significant. On the other hand, considering that the structural and functional organization of the adult brain is for the most part laid down during pregnancy and the early postnatal years and we largely have to deal with these for the rest of our lives, it is plausible that aberrations in these early processes make the brain more vulnerable for disease in later life. The present review focuses on the concept of reserve capacity of the brain and the proposition that factors playing during the earliest stages of life largely determine the reserve capacity of the brain thereby influencing the degree to which individuals are clinically affected by neuropathology in the brain in later life.

## Brain and Cognitive Reserve

### The Concept of Reserve Capacity

With increasing age, many people are faced with cognitive decline, and some individuals go on to develop a form of dementia eventually. Although neuropathological signs of dementia disorders can often be demonstrated in brains of patients, the degree to which clinical symptoms are present does mostly not show a one-to-one relation with the amount of neuropathology present. An illustrative example of this has been provided by the 100-plus Study, which is a prospective cohort study of centenarians in the Netherlands who were cognitively healthy during inclusion into the study (Beker et al., 2021). Postmortem observations in cohort members revealed that the centenarians were not without neuropathology, but showed varying loads of amyloid-β, neurofibrillary tangles, and neuritic plaques, which were however not associated with cognitive performance or decline (Beker et al., 2021). This lack of association between neuropathology in the brain and clinical symptoms of cognitive deterioration can be explained by the concept of reserve capacity. The reserve model contends that individuals differ in reserve capacity, which was originally defined as the ability of the brain to effectively buffer the changes associated with normal aging and cope with pathological damage (Stern, 2009).

A distinction is usually made between two different types of reserve capacity, brain reserve (BR) and cognitive reserve (CR) capacity. These types have also been referred to as neurobiological/structural/hardware/passive versus functional/behavioral/software/active reserve capacity. BR is commonly conceived as the neurobiological capital, in the form of anatomical or structural aspects of the brain, e.g., the size of the brain and numbers of neurons, synapses, etc. (Stern et al., 2020). Clinical symptoms of disease occur after a certain fixed pathological threshold has been reached, which may vary per person (Satz, 1993). In individuals with more BR, it will typically take longer before this threshold is reached as there is more to lose. BR is mostly seen as something that is fixed, although recent research indicated small fluctuations across the lifespan (Caspi et al., 2020). However, of course with increasing age the structure of the brain may change following age-related neurodegenerative processes. To account for these age-related brain changes, the concept of brain maintenance (BM) has been called into life. BM refers to the relative absence of changes in neural resources or neuropathological change over time as a determinant of preserved cognition in older age and depends on genetic and lifestyle factors and indicates structural brain changes over time (Collaboratory on Research Definitions for Reserve and Resilience in Cognitive Aging and Dementia, 2022). CR is the cognitive flexibility in coping with aging problems. It has been defined as the adaptability of cognitive processes that helps to explain differential susceptibility of day-to-day function to brain aging, pathology, or insult (Stern et al., 2020). CR has been suggested to depend on a range of social, mental and lifestyle factors (Stern, 2012) and is nowadays operationalized as including three components, namely (1) measures of life course related brain changes, insults, disease or risk factors that theoretically impact cognitive outcomes, (2) measures of associated change in cognition, and (3) variables that influence the relationship between 1 and 2 (Collaboratory on Research Definitions for Reserve and Resilience in Cognitive Aging and Dementia, 2022). These variables may include the highest achieved educational level, IQ, occupation, physical activity, leisure activities, and social engagement.

### Markers of Brain Reserve and Cognitive Decline and Dementia

A detailed overview of studies examining markers of brain and cognitive reserve in relation to cognitive decline and dementia disorders is out the scope of the present study. However, a short impression of the existing literature illustrates the role of reserve as a potential buffer to the development of neurodegenerative disorders. Intracranial volume seems a good proxy for brain reserve as it reflects the maximum attained brain size, but its quantification requires MRI scanning, making it an expensive and time-consuming measure to collect. Smaller ICV has been associated with higher risk for mild cognitive impairment, dementia and earlier age of onset of Alzheimer’s Disease (Schofield et al., 1995; Wolf et al., 2004; Shenkin et al., 2009). A recent meta-analysis showed that intracranial volume was positively associated with cognitive functioning when controlling for neuropathology (van Loenhoud et al., 2018). Head circumference provides a more rough measure of maximal attained brain size showing moderate correlations with intracranial volume, but is more easily obtained and has therefore more frequently been investigated in relation to cognitive function and dementia. A smaller head circumference in adult life has been associated with a higher prevalence of dementia and with the severity of cognitive impairment in Alzheimer’s Disease (Graves et al., 1996; Schofield et al., 1997; Mortimer et al., 2003; Kim et al., 2008; Perneczky et al., 2010).

### Markers of Cognitive Reserve and Cognitive Decline and Dementia

Variables such as the highest achieved educational level, occupation, physical activity, leisure activities, and social engagement have been suggested to contribute to CR and may serve as proxy measures. A meta-analysis of 15 prospective cohort studies showed that dementia risk was reduced by 7% per year increase in education (Xu et al., 2016). Higher physical activity levels have also been associated with a reduced risk of development of dementia in a meta-analytical study (Beydoun et al., 2014). The same study showed positive evidence for diet factors protecting against dementia. Interestingly, in particular the combination of the various CR factors seems strongly associated with risk for dementia. A study in which the data of 29,000 individuals was combined in a meta-analysis and cognitive reserve was operationlized as the sum of education, occupation, pre-morbid IQ, and mentally stimulating leisure activity demonstrated that men and women with a high reserve had a 50% lower chance of developing dementia over a follow-up period of 7 years (Valenzuela and Sachdev, 2006). The authors of this study advised that future research should focus on increasing our neurobiological understanding of the reserve effect in humans. A better understanding of reserve would in the future allow us to potentially manipulate the reserve and use its buffering effect on dementia incidence as a preventive strategy. An important part of the key to this may lie in early life.

## Developmental Origins of Health and Disease and Neurodegeneration

The Developmental Origins of Health and Disease hypothesis proposes that factors playing during the earliest phases of life, from pre-conception to around 2 years of age (first 1,000 days), may program the structure and physiology of the fetus/child, thereby influencing the risk for development of disease in later life (Gluckman and Hanson, 2006). The Developmental Origins of Health and Disease concept has come a long way since the landmark studies by David Barker and consorts demonstrating clear associations between small size at birth and increased risk for diabetes and cardiovascular disease in later life (Barker, 2007). By now, developmental origins of many mental and physical disorders have been established and the importance of beneficial conditions during the first 1,000 days is increasingly being recognized and acted upon by medical staff as well as public health organizations and governments (Suzuki, 2018). Still, there remains a lot to learn and the potential role of early life factors in neurodegenerative diseases has received relatively little attention. This while the brain is an organ that seems particularly sensitive to early life influences.

The development of the human brain begins *in utero* and continues through adolescence and early adulthood (Ernst and Korelitz, 2009). However, the largest part of neurogenesis is completed about halfway through gestation. Throughout the course of pregnancy, the fetal brain produces something in the order of an astonishingly 250,000 neurons (Institute of Medicine and National Academy of Sciences, 1992). Before the child has reached school age, over 95% of the maximal brain size has been achieved (Dekaban and Sadowsky, 1978). During this critical developmental phase, the brain is vulnerable to environmental influences, such as stress or undernutrition (National Research Council, Institute of Medicine, and Committee on Integrating the Science of Early Childhood Development, 2000). The brain may adapt to these circumstances and this adaptation process is usually beneficial from an evolutionary point of perspective because it provides the organism with an opportunity to adapt itself to the expected environment within a single generation (Gluckman et al., 2005). This developmental plasticity stands in contrast to genetic adaptation which takes much longer than a single generation to be effective. However, not all responses to adverse factors will be adaptive. When faced with a lack of resources for example, the organism may need to make trade-offs in order to simply survive, and this may result in developmental disruption. Although beneficial on the short-term, the permanent modifications in organ structure or physiology that the fetus/child imposes to face environmental challenges may turn out to have a negative impact in later life resulting in increased risk for disease. The brain has been put forward as a particularly prominent target for developmental programming processes. Next to being highly plastic during these developmental stages, the brain also requires cues from its environment to develop properly (Ismail et al., 2017). This makes the developing brain highly vulnerable to adverse early life circumstances.

Evidence for the Developmental Origins of Health and Disease concept has been abundant with respect to somatic diseases like cardiovascular disease and type 2 diabetes and mental health diseases such as depression (Abdul-Hussein et al., 2021; Fleming et al., 2021; Hoffman et al., 2021). Numerous studies have demonstrated that small size at birth, a marker for an adverse fetal environment, as well as adverse conditions like maternal smoking, drug use, undernutrition, overnutrition, stress, mental health problems, and toxin exposure can increase the risk for disease in later life. Although studied much less often, there is also evidence that early life factors play a role in cognitive decline and dementia disorders (Borenstein et al., 2006; Seifan et al., 2015). The Helsinki Birth Cohort study showed that in men who underwent cognitive testing at around 20 years of age and again at 68 years, small size at birth was associated with worse cognitive abilities at 20 and 68 and also with more cognitive decline (Raikkonen et al., 2013). A study in 4,000 twins in Sweden showed that infants with smaller size at birth, either low birth weight or small head circumference, were at higher risk for age-related cognitive dysfunction compared to those with normal growth (Mosing et al., 2018). Studies on late life consequences of prenatal famine exposure showed that undernutrition during gestation was associated with poorer cognitive function and more cognitive decline in late life as well as an increased risk for dementia (de Rooij et al., 2010; Kang et al., 2017; Rong et al., 2019). A number of studies has demonstrated that place of birth as a reflection of the early life environment is related to dementia, showing an increased risk for those born in less optimal circumstances. For example, people born in rural compared to urban areas and those born in areas with high risk for infant mortality or high risk for mortality due to stroke had an increased risk for dementia (Baker et al., 1993; Jean et al., 1996; Scazufca et al., 2008; Glymour et al., 2011; Gilsanz et al., 2017). Living in a rural area during childhood has also been associated with a higher risk for reduced cognitive functioning in later life (Herd et al., 2021). Poorer socio-economic conditions during early life have frequently been observed to be associated with late life cognitive impairment and increased risk for dementia especially when additional risk factors such as a family history of dementia or APOE-E4 carriership were present (Mortimer et al., 1998; Moceri et al., 2001; Zhang et al., 2008). Another factor that seems to elevate the risk for dementia is the experience of adverse events during childhood. Losing a parent at young age increases dementia risk (Norton et al., 2011; Conde-Sala and Garre-Olmo, 2020). A large Japanese study showed that people who had experienced three or more adverse childhood events had a doubled risk of developing dementia compared to those who had not experienced adverse events (Tani et al., 2020).

The early life factors and exposures demonstrated to be associated with cognitive decline and dementia in later life are diverse in nature, but may lead to dementia via similar pathways. Reserve capacity is largely built during early life and adverse early life factors and exposures may negatively affect the building of reserve capacity.

## Early Life Factors and Markers of Brain Reserve

As described above, brain reserve capacity may be represented by the size of the brain and the numbers of neurons and synapses. The maximal attained brain size is often operationalized by the intracranial volume. It is dependent on growth of the brain and usually reaches its maximum size at around 7 years of age (Rushton and Ankney, 1996). The intracranial volume is closely related to brain volume in early life but less so in later life as the brain loses volume due to age-related atrophy (Hedman et al., 2012). Intracranial volume is for a large part determined by genetic factors but early environmental factors are very likely to also play a role (Adams et al., 2016). Normal brain development requires oxygen, nutrition (energy, proteins, and micronutrients), sensory stimulation and social interaction (National Research Council, Institute of Medicine, and Committee on Integrating the Science of Early Childhood Development, 2000). Aberrations in the environment may thus cause disruptions in brain development potentially influencing growth and the maximum attained size of the brain. A clear example of this has been provided by the Dutch famine birth cohort study (de Rooij et al., 2010). Sixty-eight year old men, who had been *in utero* at the time of a 5-month famine that struck the western part of the Netherlands toward the end of World War II, appeared to have smaller intracranial volume (about 5%) compared to unexposed men. This was shown by an MRI study in the cohort aimed at investigating the long-term consequences of prenatal exposure to undernutrition for the brain. This study was unique in showing associations between an exposure during fetal life and a measurement of intracranial volume almost 7 decades later. However, there have been many studies providing evidence for associations between early life exposures and intracranial volume within a shorter time span. Women who were pregnant during the destruction of the World Trade Centre (WTC) on 11 September, 2001 and who consequently suffered from posttraumatic stress syndrome, gave birth to babies with smaller head circumference (Engel et al., 2005). A large population based Dutch birth cohort study has shown positive relationships between maternal folate levels during pregnancy and intracranial volume at the age of 9–11 years (Zou et al., 2021a). The study also showed positive associations between maternal polyunsaturated fatty acids during pregnancy and total gray and white matter volumes at the same age as well as a negative association between persistently high levels of maternal depressive symptoms across the perinatal period and total gray and white matter volumes (Zou et al., 2019; Zou et al., 2021b). Alcohol consumption during pregnancy, especially heavy use of alcohol, has been related with smaller intracranial volume in late childhood in several studies (Willoughby et al., 2008; Nardelli et al., 2011; Roussotte et al., 2012). Smaller intracranial volumes were also seen in children whose mothers took antiepileptic drugs during pregnancy (Sreedharan et al., 2018). Although intracranial volume as a specific outcome has not been investigated in studies on the environment that young children grow up in, undernutrition during the 1st year of life, the environment and SES in (early) childhood have been related to brain volumes in (later) childhood underscoring the importance of the early childhood period for brain development (Ivanovic et al., 2000; McDermott et al., 2019; Taylor et al., 2020; Hackman et al., 2021).

Small size at birth as an indirect measure of more adverse fetal circumstances has also been shown to predict brain volumes in late life. Low birth weight relative to birth length has been shown to be associated with lower brain volumes at 75 as well as reduced processing speed and executive function (Muller et al., 2014). A recent study among elderly in their early seventies demonstrated that birth weight was positively associated with total brain volume and regional cortical surface area amongst others, which could be explained by larger intracranial volume, rather than by age-related tissue atrophy (Wheater et al., 2021).

Early life factors in relation to adult head circumference have been little studied but various studies have shown associations between a diversity of prenatal exposures and head circumference at birth. For example, antenatal corticosteroid exposure was related with smaller head size at birth, as were exposure to the Zika virus, maternal psychological distress during pregnancy, maternal smoking and second hand smoking exposure, and pesticide exposure (Thorp et al., 2002; Zhou et al., 2014; Kang et al., 2017; Soesanti et al., 2019; Cranston et al., 2020; MacGinty et al., 2020).

The presented review of studies above is not meant as an exhaustive overview of the literature, but the evidence that is provided by these studies strongly suggests that the prenatal and early postnatal environment affect the development of the brain and together with genetic factors determine the ultimate size that the brain will be able to grow into thereby establishing the capacity of the brain reserve.

## Early Life Factors and Markers of Cognitive Reserve

Most studies examining CR have operationalized CR based upon (a combination of) number of years of education, IQ, and occupation. The early life environment is increasingly being acknowledged as a highly essential determinant of educational attainment and type of occupation individuals achieve. As with BR, there is evidence for prenatal and childhood exposures as well as small size at birth in relation to educational level, IQ, and type of occupation.

Several prenatal exposures have been negatively related to IQ, mainly in children, with exposures including amongst others endocrine disrupting chemicals, paracetamol, a mixture of heavy metals, pesticides and phenols, phthalates, and alcohol and smoking (Kodituwakku, 2007; Herrmann et al., 2008; Ejaredar et al., 2015; Bauer et al., 2018; Guo et al., 2020; Tanner et al., 2020). Studies on long term effects of prenatal exposures have examined educational and occupational attainment. A study on long-term consequences of malaria exposure during prenatal or early postnatal life showed that exposure was related with considerably lower levels of educational attainment and higher rates of poverty later in life (Barreca, 2010). In a similar vein, prenatal exposure to 1,918 influenza pandemic in the United States was associated with reduced educational attainment, lower income and lower socio-economic status and higher welfare payments (Almond, 2006). Pre and postnatal exposures to famine have also been associated with less educational years and worse labor market outcomes in several studies (Neelsen and Stratmann, 2011; Jürges, 2013; Scholte et al., 2015).

Data from the Survey of Health, Aging, and Retirement in Europe demonstrated that favorable early life circumstances, and in particular a higher childhood SES, were associated with a higher level of education and a higher incidence of employment, with the latter mainly driven by attained educational level (Flores and Kalwij, 2014). Many other studies have demonstrated a link between childhood socio-economic status and educational attainment. According to a report by the global Organization for Economic Cooperation and Development, socio-economic status is associated with significant differences in performance in most countries and economies that participate in the Program for International Student Assessment, with advantaged students outscoring their disadvantaged peers by large margins (OECD, 2016). Similar evidence has been found for the relationship between socio-economic status in childhood and IQ. A longitudinal study in twins showed that children from low socio-economic status families scored on average 6 IQ points lower at age 2 than children from high socio-economic backgrounds and by age 16, this difference had almost tripled (von Stumm and Plomin, 2015). Outcomes of these studies are potentially confounded by genetic background. Interestingly, however, a recent study demonstrated independent contributions of a polygenetic risk score for educational attainment and socio-economic status to cognitive function and brain development during adolescence (Judd et al., 2020).

Childhood adversity has also been linked with more negative educational outcomes and IQ. A systematic review of studies examining educational outcomes in children who had been in contact with social welfare in England showed that these children performed much worse than their peers on all outcomes measured: exam results, absences, exclusions, school moves, being missing from school, higher education aspirations, and quality of school (Jay and McGrath-Lone, 2019). Studies also found lower IQ scores in children who had been exposed to violence or harsh parenting (Delaney-Black et al., 2002; Holland et al., 2020). A study in the US demonstrated that adults with multiple adverse childhood experiences were more likely to report high school non-completion, unemployment, and living in a household below the federal poverty level compared to adults without adverse childhood experiences (Metzler et al., 2017).

A large body of evidence exists relating being born small for gestational age as compared to being born appropriate for gestational age to intellectual performance. A review on this topic describes evidence of being small for gestational age to be associated with a lower IQ at preschool age, school age and in adulthood as well as with lower academic achievement, a lower likelihood to have professional or managerial jobs and a higher likelihood of work as an unskilled, semiskilled or manual laborer resulting in a lower income (Lundgren and Tuvemo, 2008). In a very large Swedish study in 276,000 18-year old conscripts low birth weight, short birth length, small head circumference at birth and preterm birth were all associated with an increased risk for subnormal performance on a standardized IQ test (Lundgren et al., 2003).

There is thus a substantial evidence base for a role of the early environment, *in utero* and in early childhood, in shaping the most essential factors that may contribute to CR.

## Early Life Factors and Brain Maintenance

BM indicates structural brain changes over time, mainly the preservation of brain integrity in older age by buffering against neuronal loss but potentially also by regulation of adult neurogenesis (Mu and Gage, 2011). Lifestyle has been shown to play a meaningful role in how our brains physically change with aging, with several studies having shown that physical activity and diet have positive effects on various indicators of brain health (Cabral et al., 2019; Chen et al., 2019; Rodrigues et al., 2020).

Interestingly, there is evidence suggesting that lifestyle choices are influenced by early life factors. In this way, the risk for neurodegenerative diseases may not only directly but also indirectly be negatively or positively affected by the early environment via effects on levels of physical activity and dietary preferences. A highly illustrative example of this is a study among individuals who were born around the time of the Dutch famine which showed that those who had been exposed to undernutrition in early gestation tended to be less physically active and showed a preference for a high fat diet at the age of 58 years (Lussana et al., 2008). The group of exposed individuals was also shown to be at higher risk for dyslipidemia, diabetes, and coronary heart disease and to have worse cognitive function at 58 and increased brain age at age 68 (De Rooij et al., 2021). Some studies have demonstrated that both birth weight at the very low end and birth weight at the very high end are associated with lower levels of physical activity in later life (van Deutekom et al., 2017). A higher birth weight has been related to consuming a less healthy diet in childhood, while low birth weight in boys has been associated with a higher intake of fats at school age (Bischoff et al., 2018; Eloranta et al., 2018). There are few studies available that have investigated early postnatal factors in relation to late life physical activity and dietary preferences in humans but evidence that these preferences are influenced by the early environment from animal experimental studies is overwhelming (Gardner and Rhodes, 2009). These effects seem to be caused by altered wiring of the hypothalamic circuits that regulate energy balance and epigenetic changes including altered DNA methylation of key adipokines such as leptin (Reynolds et al., 2015).

The evidence discussed here is thus pointing at an indirect route from adverse early life circumstances to an increased risk for neurodegenerative diseases via its negative impact on unhealthy lifestyle choices thereby negatively affecting potential for BM.

## Discussion

### Potential Mechanisms Underlying Developmental Effects on Brain and Cognitive Reserve

There are several ways in which early life factors can impact brain and cognitive reserve. Early life factors likely affect brain development via a combination of different (related) mechanisms and these may depend on the specific factor or combination of multiple factors and timing of exposure. Evidence for these mechanisms mainly comes from experimental research in which animals are deprived in some way during pre-, peri-, or early postnatal life. A short overview of mechanisms is given below.

#### Shortage of Building Blocks

For the brain to develop it needs oxygen, energy and proteins, but also micronutrients and fatty acids. A lack or disturbance in availability of any of these factors may be caused by different conditions (undernutrition, malnutrition, environmental exposures, and stress) and may hamper brain growth and differentiation directly. A good example of this is constituted by folate. It is well known that sufficient levels of folate are necessary to prevent neural tube defects such as spina bifida and anencephaly. Smaller variations in folate levels however, may also have detectable effects such as shown by the already referred to study that demonstrated an association between maternal folate levels during pregnancy and intracranial volume at the age of 9–11 years (Zou et al., 2021a).

#### Energy Metabolism

Early life factors may also program biological regulatory processes, such as energy metabolism in the brain, which then affects growth, differentiation, and function. Experiments in rats, for example, have shown that a low protein diet during pregnancy and lactation induced long-lasting changes in nutrient sensing and energy homeostasis in the hypothalamus, which likely affects its functioning (Orozco-Solis et al., 2010). Specifically, changes in mitochondrial function may alter energy metabolism. Mitochondrial dysfunction has been demonstrated to be a central developmental programming pathway affecting many different tissues (Ozanne, 2014). Considering that the brain is highly vulnerable for mitochondrial dysfunction as it requires large amounts of energy during its development as well as in adult state, this may have profound effects on brain development and function, also in later life (Morava and Kozicz, 2013).

#### Neural Plasticity

Another and related mechanism underlying developmental effects on BR and CR is programming of neural plasticity. Neurotrophic factors are a family of biomolecules that support the growth, survival, and differentiation of both developing and mature neurons and play an important role in neuroplasticity (Cohen and Greenberg, 2008). They are linked with mitochondrial function in the brain, as well as with dementia disorders (Markham et al., 2014; Budni et al., 2015). Evidence from studies in baboons demonstrated clear effects of maternal undernutrition on neurotrophic factors in fetal baboons and they also showed effects on later brain age (Antonow-Schlorke et al., 2011; Franke et al., 2017).

#### Cortisol

Stress exposure activates the Hypothalamic-Pituitary-Adrenal (HPA)-axis which results in the secretion of cortisol and is a frequently studied pathway from early life adversity to altered brain development. Direct exposure to elevated levels of cortisol seems to affect brain development as suggested by a study in youth where pre bedtime cortisol levels were negatively associated with prefrontal cortex gray matter volumes (Carrion et al., 2010). Long-term consequences have also been demonstrated, for example by rodent studies that showed that prenatal stress induces cognitive impairments in late life which are accompanied by life-long changes in the HPA-axis (Koehl et al., 2001).

#### Brain Circuit Maturation

Early life factors may also have profound effects on brain circuit maturation (Short and Baram, 2019). Complex cognitive functions depend on structural connectivity between brain circuits and the maturation of these is likely negatively influenced by adverse early life conditions. This is illustrated by development of hippocampal formation circuits, which undergoes dramatic growth and maturation during the early postnatal period [see Short and Baram (2019)]. Studies have found excessive loss of hippocampal dendrites, dendritic spines and synapses during this period in rodents exposed to early life adversity (Short and Baram, 2019).

#### Epigenetic Mechanisms

An overarching mechanism that may underlie both structural and functional adaptations in response to early life conditions is altered gene expression through epigenetic processes including DNA methylation. DNA methylation differences have frequently been observed in blood after early life adversity in humans, and similar methylation differences have also been shown in the brains of mice (Provençal and Binder, 2015; Catale et al., 2020).

Other mechanisms have been put forward, such as alterations and programming of immune factors and other hormones than cortisol, such as insulin, leptin, and sex hormones. The large number of different mechanisms that have been proposed to underlie associations between early life factors and brain development and function illustrate the complexity as well as the far reaching multitude of potential effects that these factors may have on the brain as a whole on the short term but also on the long-term.

### Evidence Synthesis and Discussion

Taking all evidence together, we can deduce that a poor early start in life may lead to a smaller brain and cognitive reserve capacity and a smaller potential to maintain reserve, limiting the capability to buffer effects of age-related neuropathology resulting in a higher risk for developing dementia disorders in later life. A poor early start can be caused by many different factors at play during roughly the first 1,000 days of life. A large number of prenatal factors have been shown to relate to indicators of BR, CR, and BM, including maternal shortage of macro and micronutrients, maternal stress and depression, substance use and medication, exposure to chemicals, air pollution and heavy metals and small size at birth. Early postnatal factors, during the first 2 years of life, have been less frequently studied in relation to BR, CR, and BM indicators and have mainly focused on SES and poor living circumstances in early childhood with substantial evidence for associations between these factors and indicators such as lower IQ and lower educational and occupational attainment. Often, prenatal factors will overlap with postnatal factors and several different (related) factors may play at the same time.

The influence of the early life environment on reserve capacity is supposedly direct by affecting BR, CR, and BM factors. Early life factors influence growth and differentiation and in this way, together with genetic factors, establish BR (Adams et al., 2016). Early life factors also impact IQ, educational and occupational attainment contributing to CR. Keeping BR and CR up at later age is supported by BM factors such as a healthy diet and sufficient levels of physical activity, health behaviors that are also programmed by early life factors. On top of this, the impact of early life environment on reserve capacity is also indirect, as indicators of BR such as intracranial volume and head circumference at birth as well as later on are determinants of IQ and educational attainment contributing to CR (Adams et al., 2016). This was demonstrated by a study in which MRI data were collected from young adults who had been exposed to severe deprivation in early childhood in the Romanian orphanages of the Ceauşescu era and were then adopted by United Kingdom families (Mackes et al., 2020). These young adults were shown to have overall smaller brain volume which mediated the observed relationship between institutionalization and a lower IQ.

In conclusion, the early life environment likely has a substantial role in determining reserve capacity and the potential to maintain it, which extends years beyond the initial exposure. However, some side notes must be made. First of all, studies on associations between early life factors and indictors of BR, CR, and BM may be confounded by genetic effects or variables that are related to both the early environment and later brain health outcomes, such as a common place where people are born and reside in later life. Studies where the long-term consequences of early life exposure to natural disasters or war on brain health have been investigated, however, do provide clues on causality. Individuals who had been prenatally exposed to the 1944–1945 Dutch famine, which struck all social classes, had worse cognitive function, smaller intracranial volume and older appearing brain in late life compared to their contemporaries who had been born just before the famine or had been conceived after it (De Rooij et al., 2021). The quasi experimental nature of this study suggests no confounding by genetics or other variables (De Rooij et al., 2021).

Secondly, modulation of reserve capacity is not the only pathway by which early life factors may program cognitive health and risk for dementia disorders in later life. Evidence from animal studies suggests that an adverse early environment may expedite the pathological processes underlying dementia disorders and vice versa. In a transgenic mouse model which progressively develops combined tau and amyloid pathology, mice were subjected to either early life stress or to ‘positive’ early handling postnatally (from day 2 to 9). The mice subjected to early life stress displayed increased hippocampal amyloid-beta levels at 4 months of age, while these levels were reduced in the mice subjected to early handling, which also lived longer (Lesuis et al., 2016). Several molecular pathways have been suggested that may link early life adversity to an increase in later life neuropathology, including programming of the HPA-axis, programming of the neuroinflammatory response, dendritic and synaptic complexity and function, overall brain plasticity, and some proteins (Lesuis et al., 2018). Another route from early life adversity to dementia disorders is through the increased risk of hypertension, diabetes and cardiovascular disease, diseases that all have been demonstrated to have developmental origins and which increase the risk for dementia disorders.

Thirdly, there is evidence that neurogenesis (the formation of new neurons) and synaptogenesis (the formation of synapses) occur in the adult brain, whereas this was thought to be non-existent years ago (Kempermann et al., 2018). Although still controversial, this could mean that BR could potentially increase at adult age. The same factors that influence BM are thought to be at play here, so factors like diet, exercise and social interactions. There is especially strong pre-clinical evidence for physical activity as an important modulator of neurogenesis as well as synaptogenesis, mirrored by some evidence in humans (Ambrogini et al., 2013; Phillips, 2017). Considering the evidence that the early environment can program physical activity levels in later life, this seems yet another route by which early life factors may impact BR.

Finally, a serious limitation to research on reserve capacity is the fact that we have to rely on supposed indicators of reserve capacity. Reserve capacity is theorized to be able to sustain cognitive function against a certain level of accumulated brain damage or degeneration. To actually know the level of reserve capacity, we would need to know both the level of cognitive functioning as the level of neuropathology at the some point in time. Whereas we can measure the first, we cannot measure the latter (yet). Researchers are currently trying to develop better measures of CR based on brain networks and functional connectivity within the brain assessing the capacity to efficiently process information (Franzmeier et al., 2017; Serra et al., 2017; Lee et al., 2019). Initiatives such as these will help in better measurement of reserve capacity and studying the influence of early life factors on the reserve capacity.

### Relevance and Future Directions

Research on long-term associations between early life risk factors and risk for dementia disorders is constrained by the long time lag between exposure and outcome. Studies that have investigated this association are largely retrospective by nature and thus hampered by the limitations of this study design (i.e., retrospective ascertainment of exposure, selective participation of more healthy subjects, and recall bias). Given that the Developmental Origins of Health and Disease field of research is a relatively young research area, many early life factors that may be significant in relation to cognitive decline and dementia disorders have not yet been investigated as such. Based on the evidence presented here, showing that a diversity of early life factors may influence BR and CR as well as BM potential, it may be expected that a wide range of early life factors, both prenatal and postnatal, affects the risk for dementia disorders.

Besides the lack of investigation of many other early life factors in relation to BR and CR, there are a number of topics that have not been touched upon yet or that have been rarely studied only in this field of research. Intracranial volume and head circumference are the main indicators of BR that have been investigated in relation to early life factors. However, measures such as cortical thickness and white matter microstructure are also valuable indicators of BR, but have been little studied in relation to early life factors, especially at later stages of life. Also, the majority of studies investigating relationships between early life factors and later life brain and cognitive health indicators have examined single early factors. Outstanding questions are whether these early life factors all contribute independently to BR and CR or whether they account for the same variance and moreover if these factors interact with each other in contributing to BR and CR. In addition, early life factors have mainly been associated with indicators of BR and CR as determinants of these separate concepts, but these factors may potentially act as moderators of the relationship between levels of pathology and cognitive functioning and should be studied as such. Studies aimed at answering these open questions are needed.

We have seen that adverse circumstances may impact the developing brain in a negative way, but the opposite may also hold. The early life period presents a window during which damaging environmental conditions may have devastating effects on brain development, but during which preventive efforts may also have the greatest benefits. In other words, the evidence presented here also provides us potential directions on how to promote the building of reserve capacity. In 2017, a paper was published by the Lancet Commission on Dementia Prevention, Intervention, and Care promoting a broader approach to prevention of dementia (Livingston et al., 2017). In this statement, the authors advocated an approach whereby cognitive resilience in later life is enhanced by building brain reserve earlier in life, focusing on socio-economic status in early life and education. However, the studies discussed in the present review demonstrate that a very wide range of early life factors may influence reserve, including early life nutrition, stress, and a multitude of environmental exposures during pregnancy and early postnatal life.

Building brain reserve early in life may be achieved by two strategies. First, we can aim to provide a good start for children during the first 1,000 days. A healthy lifestyle during pregnancy and the early postnatal period, with a healthy diet, physical activity, and abstinence from smoking, drugs, and alcohol consumption is recommended as is a supportive social environment. However, adverse conditions such as high psychological stress exposure, maternal or paternal depression, financial stresses or exposure to toxic substances or air pollution is much more difficult to tackle and may not be avoided. A second strategy may consist of intervention during these adverse circumstances, which would also enable assessment of causality. Results from animal studies indicate that alterations by adverse early life circumstances are not set in stone and may be reversible. An experiment in mice demonstrated that early postnatal stress exposure led to impaired cognitive function in adult life, but feeding the young stressed mice with a diet rich in methionine and B vitamins prevented these effects (Naninck et al., 2017). In addition, a positive early postnatal environment (in the form of postnatal handling) was shown to counteract the negative effects of prenatal stress on age-related cognitive changes in rats (Vallée et al., 1999). Findings from these animal experimental studies have to be translated to humans in order to find out whether similar results can be achieved in children who experience adverse conditions in early life. A good example of this is a pilot randomized control study that was set up among pregnant women with a diagnosed clinical depression (Bleker et al., 2019). Half of the group received a specifically designed cognitive behavioral therapy program and the others received care as usual. We conducted MRI scans in the 6-year old offspring and found some structures including the right lateral occipital cortex and lingual gyrus to be thicker in children whose mother had underwent antenatal treatment. Measuring potential longer-term effects of such interventions in adverse early life factors on indicators of reserve capacity, such as intracranial volume, head circumference, IQ and education would learn us what we can do to boost the potential of the brain to mitigate effects of neurodegeneration in later life and thereby decrease the risk for developing dementia disorders.

Finally, but importantly, as discussed above, BM is influenced by lifestyle factors. Physical activity, a healthy diet, social contacts and continuing to stimulate the brain with learning new things may be extra important for individuals who have exprienced an adverse early life environment.

## Author Contributions

The author confirms being the sole contributor of this work and has approved it for publication.

## Conflict of Interest

The author declares that the research was conducted in the absence of any commercial or financial relationships that could be construed as a potential conflict of interest.

## Publisher’s Note

All claims expressed in this article are solely those of the authors and do not necessarily represent those of their affiliated organizations, or those of the publisher, the editors and the reviewers. Any product that may be evaluated in this article, or claim that may be made by its manufacturer, is not guaranteed or endorsed by the publisher.
